# Integrated Analysis of Transcriptome and Metabolome Provides Insights into Flavonoid Biosynthesis of Blueberry Leaves in Response to Drought Stress

**DOI:** 10.3390/ijms252011135

**Published:** 2024-10-17

**Authors:** Xinghua Feng, Sining Bai, Lianxia Zhou, Yan Song, Sijin Jia, Qingxun Guo, Chunyu Zhang

**Affiliations:** 1 Department of Horticulture, College of Plant Science, Jilin University, Changchun 130062, China; 2 Jilin Engineering Research Center for Crop Biotechnology Breeding, College of Plant Science, Jilin University, Changchun 130062, China

**Keywords:** blueberry, metabolome, transcriptome, drought stress, flavonoids

## Abstract

Blueberries (*Vaccinium* spp.) are extremely sensitive to drought stress. Flavonoids are crucial secondary metabolites that possess the ability to withstand drought stress. Therefore, improving the drought resistance of blueberries by increasing the flavonoid content is crucial for the development of the blueberry industry. To explore the underlying molecular mechanism of blueberry in adaptation to drought stress, we performed an integrated analysis of the metabolome and transcriptome of blueberry leaves under drought stress. We found that the most enriched drought-responsive genes are mainly involved in flavonoid biosynthesis and plant hormone signal transduction pathways based on transcriptome data and the main drought-responsive metabolites come from the flavonoid class based on metabolome data. The *UDP-glucose flavonoid 3-O-glucosyl transferase* (*UFGT*), *flavonol synthase* (*FLS*), and *anthocyanidin reductase* (*ANR-2*) genes may be the key genes for the accumulation of anthocyanins, flavonols, and flavans in response to drought stress in blueberry leaves, respectively. Delphinidin 3-glucoside and delphinidin-3-O-glucoside chloride may be the most important drought-responsive flavonoid metabolites. *VcMYB1*, *VcMYBPA1*, *MYBPA1.2*, and *MYBPA2.1* might be responsible for drought-induced flavonoid biosynthesis and *VcMYB14, MYB14, MYB102*, *and MYB108* may be responsible for blueberry leaf drought tolerance. ABA responsive elements binding factor (*ABF*) genes, *MYB* genes, *bHLH* genes, and flavonoid biosynthetic genes might form a regulatory network to regulate drought-induced accumulation of flavonoid metabolites in blueberry leaves. Our study provides a useful reference for breeding drought-resistant blueberry varieties.

## 1. Introduction

Blueberry (*Vaccinium corymbosum*) is a perennial shrub known worldwide for its health benefits due to its abundant multiple bioactive substances. In recent years, increasing demand for blueberry has led to a global expansion of blueberry cultivation [1,2]. However, blueberries are particularly vulnerable to the adverse effects of drought stress due to their superficial root system and lack of root hairs, growing roots less than 40 cm deep, which limit blueberry’s ability to intake water and nutrients from the soil, especially under drought stress conditions [3,4]. Globally, the drought disaster-affected area will increase with the rising global temperature, which seriously threatens the development of blueberry industry and the yield of blueberries has decreased by around 25–30% [5,6]. Thus, it is important to explore the molecular regulatory mechanisms of the response of blueberry to drought stress for the breeding of drought-resistant blueberry cultivars.

Drought stress has negative effects at the physiological, developmental, and molecular levels in plants, including photosynthesis inhibition and reactive oxygen species (ROS) generation [6,7,8]. Plants have also developed a variety of physiological and molecular adaptive mechanisms to tolerate drought stress or slow the rate of its impact on plant physiology. For instance, flavonoids serve as nonenzymatic systems to reduce ROS generated during drought stress, and thus improve plant acclimation to drought stress [9]. Additionally, plant hormones function as central integrators that link and re-program the complex developmental and stress adaptive signaling cascades, in which ABA plays a role at the interface between plant drought response and cellular primary metabolism [7,10].

In nature, more than 10,000 flavonoid compounds have been discovered; however, the main flavonoid compounds are flavonols, anthocyanin, flavans, and proanthocyanidins in the *Vaccinium* genus [11,12]. It has been well documented that flavonoid accumulation can enhance drought tolerance in plants [13,14,15]. Flavonoid metabolites are biosynthesized via general phenylpropanoid pathway enzymes including phenylalanine ammonia-lyase (PAL), cinnamate 4-hydroxylase (C4H), and 4-coumarate CoA ligase (4CL), transforming phenylalanine into *p*-Coumaroyl-CoA, which subsequently enters the flavonoid biosynthetic pathway. In this pathway, dihydroflavonols are biosynthesized by chalcone synthase (CHS), chalcone isomerase (CHI), flavanone 3-hydroxylase (F3H), flavonoid 3′-hydroxylase (F3′H), and flavonoid 3′5′-hydroxylase (F3′5′H). Finally, FLS catalyzes flavonol biosynthesis, while dihydroflavonol 4-reductase (DFR), anthocyanidin synthase (ANS), and UFGT catalyze anthocyanin biosynthesis, and DFR, leucoanthocyanidin reductase (LAR), and ANR are responsible for flavan biosynthesis. The genes encoding these enzymes promote flavonoid accumulation and increase drought tolerance. For example, the *EkFLS* (*Euphorbia kansui*) overexpressed in *Arabidopsis* induced the accumulation of flavonoids, which significantly enhanced the activity of peroxidase (POD) and superoxide dismutase (SOD), which can scavenge reactive oxygen species in cells to protect the plant under drought treatment [16].

Transcription factors (TFs), as multi-functional proteins, may simultaneously control numerous pathways during drought stresses in plants [17]. In the flavonoid biosynthetic pathway, TFs increase the tolerance of plants to drought stress by regulating the expression of flavonoid biosynthetic genes. For example, in *Arabidopsis*, MYB12 up-regulates the expression of *CHS* and *FLS* genes and MYB75 regulates the expression of *DFR* and *ANS* [18,19]. Overexpression of MYB12 or MYB75 in transgenic plants significantly increases the accumulation of flavonoids with strong antioxidant activity, thus enhancing tolerance to drought stresses [20,21]. In grape (*Vitis vinifera*), the overexpression of the *VvbHLH1* transcription factor resulted in the up-regulation of genes involved in flavonoid biosynthesis, increasing flavonoid accumulation and enhancing drought tolerance in transgenic *Arabidopsis* [22].

Drought stress can cause the signal transduction of various plant hormones such as abscisic acid (ABA) and gibberellic acid (GA). ABA, a stress response plant hormone, plays important roles in plant defense against stress conditions and flavonoid biosynthesis [7,23]. The ABA signal transduction pathway includes PYRABACTIN RESISTANCE 1 (PYR)/PYR1-like (PYL), type 2C protein phosphatase (PP2C), sucrose non-fermenting 1-related protein kinase 2 (SnRK2), and ABA responsive elements binding factor (ABF) family members. Of these, ABF, basic leucine zipper (bZIP) transcription factor, has been reported to regulate the expression of drought-responsive genes and enhance enzyme activity to maintain plant resistance to drought stress [24,25].

In this study, we identified differentially expressed genes (DEGs) and screened DEGs from the flavonoid biosynthetic pathway and plant hormone signal transduction pathway and differentially expressed transcription factor genes from the MYB and bHLH families in blueberry leaves in response to drought stress by transcriptome analysis. The differentially accumulated metabolites (DAMs) also were identified under drought stress by metabolome analysis. To elucidate the mechanism driving flavonoid biosynthesis in blueberry leaves under drought, we predicted the key gene that regulates the accumulation of anthocyanins, flavonols, and flavans and assembled a regulatory network encompassing ABA signal transduction pathway *ABF* genes, *MYBs*, *bHLHs*, flavonoid biosynthetic genes, and flavonoid metabolites according to Pearson correlation analysis by integrating transcriptome and metabolome data. The results presented here will broaden our understanding of drought-induced flavonoid biosynthesis and provide a basis for breeding drought-resistant blueberry varieties.

## 2. Results

### 2.1. RNA-seq Quality Assessment and Differentially Expressed Genes (DEGs) from Blueberry Leaves Under Drought Stress 

The 15 RNA-Seq libraries constructed from drought stress for 0 h (P0-1, P0-2, P0-3), 6 h (P6-1, P6-2, P6-3), 12 h (P12-1, P12-2, P12-3), 24 h (P24-1, P24-2, P24-3), and 48 h (P48-1, P48-2, P48-3) were sequenced, yielding 41,142,610–46,934,934 raw reads, 41,134,874–46,924,748 clean reads, and 6.15–7.01 Gb clean bases. The GC content of all samples ranged from 45.83% to 46.43%, the percentage of the Q20 bases was greater than 96.1%, and the percentage of Q30 bases was greater than 89.3%. The mapping rates obtained by comparison with a blueberry reference genome were greater than 90.33% (Table S1). Pearson correlation analysis showed that correlation coefficient (*r*) was greater than 0.95 for the three biological replicates within each treatment and PCA results indicated that samples were scattered between treatments and gathered within treatments (Figure S1a,b). These results implied that the sequencing quality was high, the sample selection process was reasonable, and the sequencing data met the requirements for further analyses.

**Figure 1:**
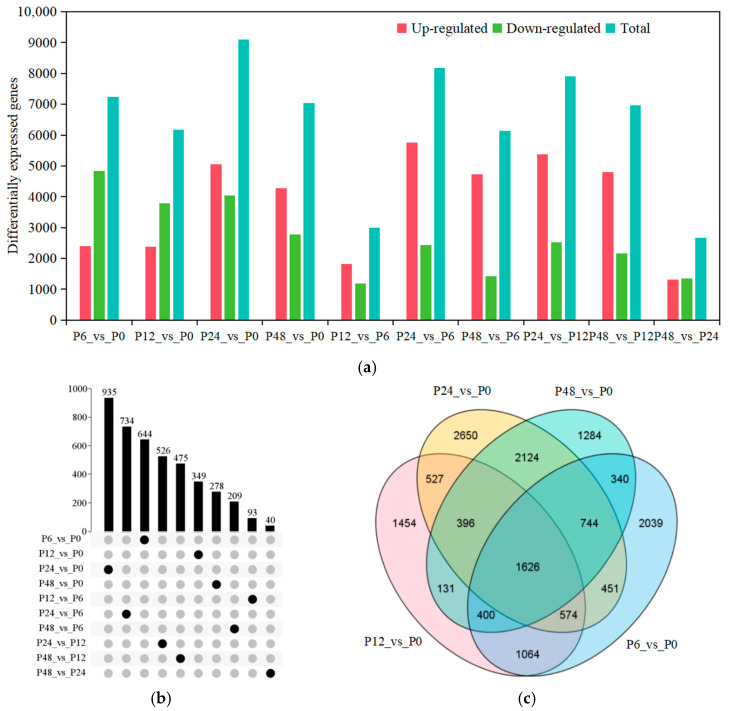
Differentially expressed genes (DEGs) in blueberry leaves in response to drought stress were identified by transcriptome deep sequencing (RNA-seq). (**a**) Number of DEGs in response to drought stress. Up-regulated, up-regulated DEGs; Down-regulated, down-regulated DEGs; Total, total DEGs. (**b**) The UpSet Plot shows the number of specific DEGs for all the pairwise comparisons. (**c**) Venn diagram showing the extent of overlap between DEGs for four pairwise comparisons. P0, P6, P12, P24, and P48 represent that 20% PEG 6000 treated the samples for 0, 6, 12, 24, and 48 h, respectively. Blue, DEGs for 6 h vs. 0 h; pink, DEGs for 12 h vs. 0 h; yellow, DEGs for 24 h vs. 0 h; green, DEGs for 48 h vs. 0 h.

To examine the effects of drought stress on gene expression, we identified DEGs during 20% PEG-6000 treatment in blueberry leaves. A total of 14,594 DEGs were detected for all ten pairwise compared groups and DEGs are listed in Table S2. The number of DEGs was the largest at P24_vs_P0 (9092), and then P24_vs_P6 (8183) and P24_vs_P12 (7901), and was the lowest at P48_vs_P24 (2667) in all the pairwise compared groups. Of these, there were more up-regulated DEGs than down-regulated DEGs, except at the early stages (P6_vs_P0 and P12_vs_P0) (Figure 1a; Table S3). The UpSet Plot showed that 935 DEGs were specific to P24_vs_P0 and 734 to P24_vs_P6 in all the pairwise compared groups (Figure 1b). These results indicated that most genes responded to drought stress at 24 h, and up-regulated DEGs increased with increasing drought treatment duration. The Venn diagram revealed that 1623 DEGs were shared among the 6, 12, 24, and 48 h of drought stress groups relative to the 0 h control (Figure 1c).

The accuracy and reliability of the RNA-seq data were validated by RT-qPCR analysis (Figure S2). A linear regression analysis showed that the RNA-seq data had a positive correlation with the RT-qPCR results, with correlation coefficients (*R*^2^) more than 0.8272 for drought stress compared to the 0 h control. This observation confirmed the accuracy and reliability of the RNA-seq data from blueberry leaves under drought stress.

### 2.2. The KEGG Pathway Analysis of DEGs Under Drought Stress

**Figure 2:**
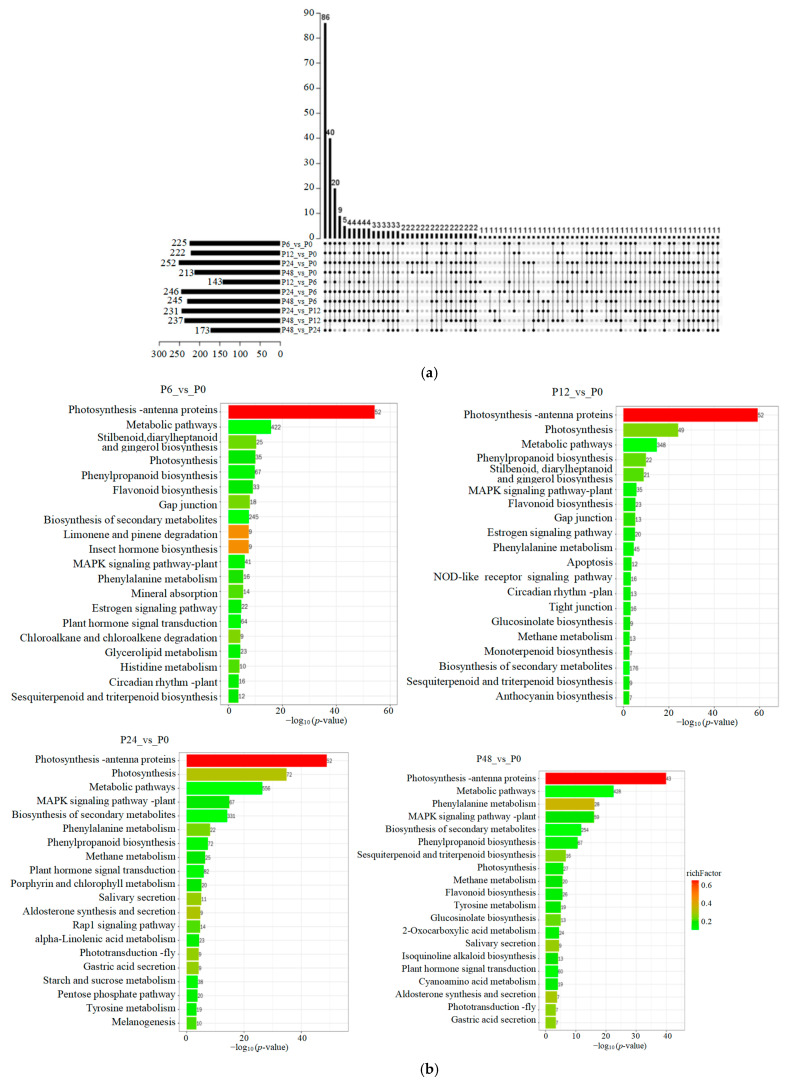
KEGG pathway enrichment analysis of differentially expressed genes (DEGs) in blueberry leaves in response to drought stress identified by transcriptome deep sequencing (RNA-seq). (**a**) The UpSet Plot of significantly enriched KEGG pathways among the DEGs. (**b**) The top 20 enriched KEGG pathways of DEGs in different comparison groups. P0, P6, P12, P24, and P48 represent that 20% PEG 6000 treated the samples for 0, 6, 12, 24, and 48 h, respectively.

The functions of DEGs under drought stress were annotated using the KEGG databases (Table S4). All the DEGs were assigned to 275 KEGG pathways, in which the DEGs from P24_vs_P0 were annotated to 252 KEGG pathways and 86 KEGG pathways were shared in all ten pairwise comparison groups according to UpSet Plot (Figure 2a). Then, we analyzed the top 20 KEGG pathways during salt stress compared to 0 h control samples (Figure 2b). We found that photosynthesis-antenna proteins (ko00196) and MAPK signaling pathway-plant (ko04016) were significantly enriched at all stages of drought treatment. Similarly, many DEGs were involved in the biosynthesis of phenylpropanoid-derived compounds, including in the phenylpropanoid biosynthetic pathway (ko00940), anthocyanin biosynthetic pathway (ko00941), flavonoid biosynthetic pathway (ko00942), and flavone and flavonol biosynthetic pathway (ko00944), and were significantly enriched at most stages, suggesting that phenylpropanoid-derived compounds were significantly enriched under drought stress. Moreover, DEGs were significantly enriched in the plant hormone signal transduction pathway (ko04075) at 6, 24, and 48 h of drought stress compared to 0 h control samples, indicating that plant hormones play important roles in the response of blueberry leaves to drought stress.

### 2.3. The DEGs of Flavonoid Biosynthetic Pathway Under Drought Stress

**Figure 3:**
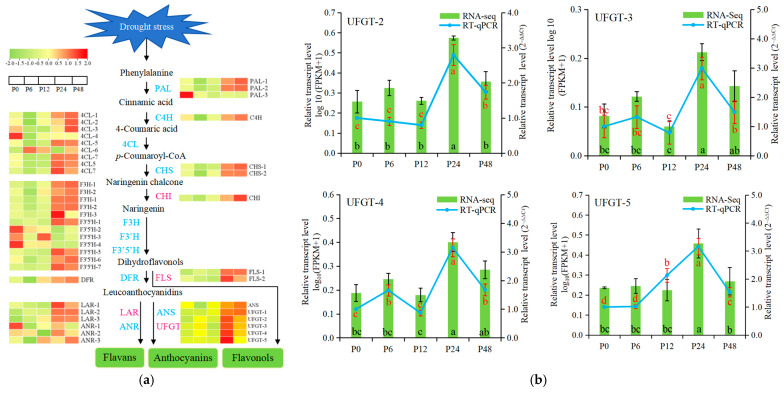

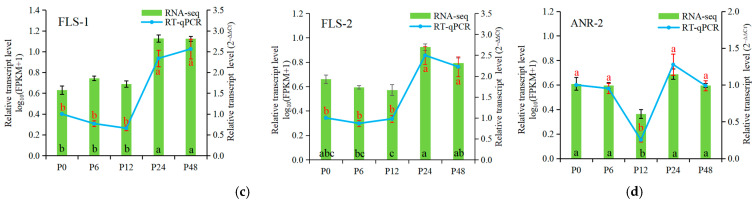
The expression of differentially expressed genes (DEGs) from flavonoid biosynthetic pathways in blueberry leaves under drought stress. (**a**) The expression of flavonoid biosynthetic genes based on log_10_ (FPKM) values from RNA-seq data. Magenta font indicates up-regulated genes and blue font indicates both up-regulated and down-regulated genes in response to drought stress. The expression patterns of *UFGT* (**b**), *FLS* (**c**), and *ANR* (**d**) genes from RNA-seq data based on log_10_ (FPKM + 1) values and RT-qPCR analysis based on 2^−∆∆Ct^ values. Values are means ± SD from three independent biological replicates. Different letters indicate significant differences between samples using *p* value ≤ 0.05. The black error bars and red error bars represent the SD of the samples for RNA-seq and RT-qPCR analysis, respectively. The black letters (a, b, c, and d) and red letters (a, b, c, and d) indicate significant differences between sample for RNA-seq and RT-qPCR analysis, respectively. P0, P6, P12, P24, and P48 represent that 20% PEG 6000 treated the samples for 0, 6, 12, 24, and 48 h, respectively.

To clarify the roles of flavonoid biosynthetic genes in response to drought stress, we identified 43 DEGs encoding 13 types of enzymes from the phenylpropanoid biosynthetic pathway, flavonoid biosynthetic pathway, flavonol biosynthetic pathway, and anthocyanin biosynthetic pathway (Table S5). These DEGs were mapped to flavonoid biosynthetic pathway (Figure 3a). The results showed that most DEGs were up-regulated under drought stress, including *4CL-2*, *4CL-5*, *4CL-7*, *4CL5*, *4CL7*, *CHI*, *F3H-1*, *F3′5′H-1*, *F3′5′H-5*, *F3′5′H-7*, *F3′H-1*, *F3′H-2*, *FLSs*, *LARs*, and *UFGTs*, and their expression significantly increased from 12 h of drought treatment and reached their highest levels at 24 h. However, other DEGs were down-regulated at the early stage (6 h) of drought stress relative to the 0 h control. Of these, the expression levels of *PAL-1*, *PAL-2*, *4CL-1*, *4CL-3*, *C4H*, *CHSs*, *DFR*, *F3H-2*, *ANR-3*, and *ANS* were up-regulated at 12 h or 24 h. At the same time, drought stress also down-regulated the expression of *PAL-3*, *4CL-4*, *4CL-6*, *F3′5′H-2*, and *F3′5′H-4*. However, most genes did not change in expression levels in the P48_vs_P24 compared group (Table S5; Figure 3a). These results indicated that expression of most flavonoid biosynthetic genes was inhibited at 6 h of drought treatment and was promoted at 24 h and 48 h of drought treatment. In general, drought stress promoted the expression of most flavonoid biosynthetic genes.

The *UFGT*, *FLS*, and *ANR* were the last-step genes of biosynthesis of anthocyanins, flavonols and flavans, respectively. We validated the expression patterns of these genes by RT-qPCR. The results showed that change trend of *UFGT-2*, *UFGT-3*, *UFGT-4*, *UFGT-5*, *FLS-1*, *FLS-2*, and *ANR-2* from RT-qPCR analysis were consistent with the RNA-seq data (Figure 3b–d). The four *UFGT* genes showed the highest expression at 24 h and two *FLS* genes showed the highest expression at 24 h and 48 h under drought stress by both RNA-seq data and RT-qPCR analysis. Both RNA-seq and RT-qPCR data also showed that the expression levels of *ANR-2* were lowest at 12 h under drought stress.

### 2.4. The DEGs of Plant Hormone Signal Transduction Pathway Under Drought Stress

**Figure 4:**
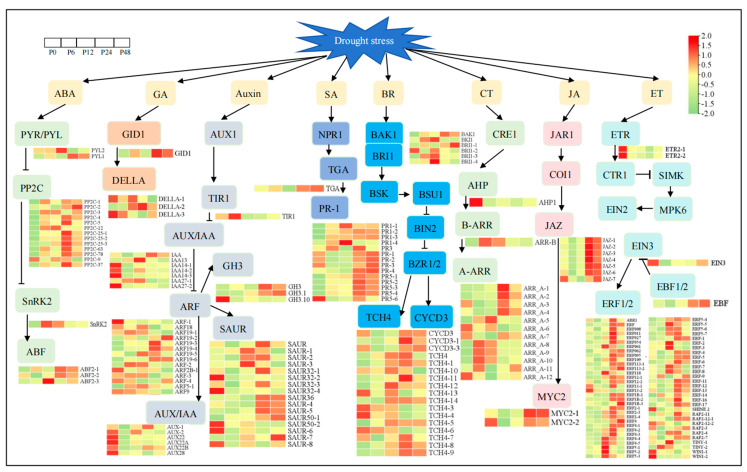
Differentially expressed genes (DEGs) from plant hormone signal transduction KEGG pathways in blueberry leaves under drought stress.

To elucidate the functions of plant hormones in response to drought stress for blueberry leaves, we screened the DEGs from the plant hormone signal transduction pathway and these DEGs were mapped to the ABA, GA, auxin, salicylic acid (SA), brassinosteroid (BR), cytokinine (CT), jasmonic acid (JA), and ethylene (ET) signal transduction pathways (Figure 4; Table S6). For GA signal transduction pathway genes, *gibberellin insensitive dwarf1* (*GID1*) was up-regulated and *DELLA*s were up-regulated or down-regulated under drought stress. For the SA signal transduction pathway, drought stress up-regulated the expression of *TGA* and most *pathogenesis-related* (*PR*) genes. The BR signal transduction pathway genes also responded to drought stress and most genes were up-regulated at 12 h and 24 h of drought treatment relative to the 0 h control. For the CT signal transduction pathway, drought stress inhibited the expression of *histidine-containing phosphotransfer protein* (*AHP*) and promoted the expression of *two-component response regulator* (*B-ARR*). Drought stress also induced the expression of *A-ARR* genes and the expression levels of most DEGs were highest at 24 h or 6 h of drought stress. For the JA signal transduction pathway, almost all the *jasmonate-ZIM domain* (*JAZ*) and *MYC2* genes had inhibited expression at 6 h or 12 h of drought stress, and then rapidly increased at 24 h and 48 h of drought stress. For the ET signal transduction pathway, drought stress down-regulated the expression of *ethylene receptor* (*ETR*) and *ethylene insensitive 3* (*EIN3*) genes and up-regulated the expression of the *EIN3-binding F-box protein* (*EBF*) gene. At the same time, 65 *ethylene-responsive transcription factor* (*ERF*) family members had their expression induced under drought stress, indicating the importance of the ERF family in response to drought stress. For the auxin signal transduction pathway, a total of 47 DEGs from six gene families were identified. Drought stress down-regulated the expression of *transport inhibitor response 1* (*TIR1*) and most AUX/IAA family members. Further, we analyzed the *auxin response factor* (AFR) genes and found that six *ARF* genes were down-regulated and eight *ARF* genes were up-regulated under drought treatment. At the same time, drought stress also induced the expression of *indole-3-acetic acid-amido synthetase* (*GH3*) genes and *small auxin up-regulated RNA* (*SAUR*) family members, and these genes were down-regulated or up-regulated under drought stress.

**Figure 5:**
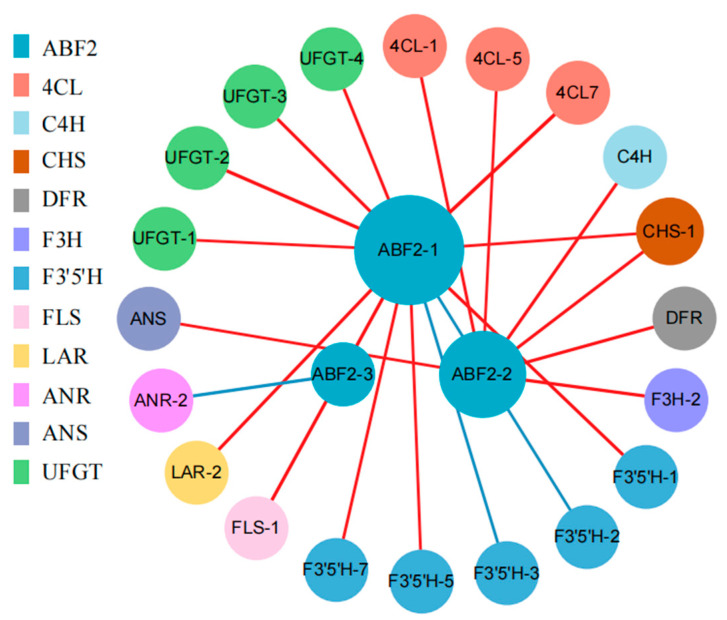
The network of Pearson correlations between differentially expressed ABF2 genes and differentially expressed flavonoid biosynthetic genes in blueberry leaves under drought stress. Red lines indicate positive correlations; blue lines indicate negative correlations. The thickness of the line represents the degree of correlation. The size of the circle represents the number of related genes. P0, P6, P12, P24, and P48 represent that 20% PEG 6000 treated the samples for 0, 6, 12, 24, and 48 h, respectively.

The nineteen DEGs from four gene families were mapped to the ABA signal transduction pathway (Figure 4; Table S6). Of these, the *PYL-1*, *SnRK2*, and most *PP2C* genes were up-regulated and the *PP2C-9* gene was down-regulated during drought treatment relative to the 0 h control. We also found that the expression of some *PP2Cs* was down-regulated for the pairwise compared groups; for example, *PP2C-1* was down-regulated at P12_vs_P6, *PP2C-3* at P24_vs_P6, and *PP2C-5*, *PP2C-25-1*, and *PP2C-25-2* at P48_vs_P24. The three *ABF2* genes responded to drought, of which *ABF2-1* was up-regulated at 6 h, 24 h, and 48 h compared to 0 h control, *ABF2-2* was also up-regulated at 24 h compared to 6 h drought treatment, and *ABF2-3* was up-regulated at 12 h compared to 6 h and down-regulated at 24 h compared to 12 h during drought stress. At the same time, we also found that the expression levels of *ABF2-1* were positively correlated with those of *4CL7*, *CHS*, *F3′5′H*, *FLS*, *LAR*, and *UFGT* genes during drought stress, and *ABF2-2* was positively correlated with *4CL*, *C4H*, *DFR*, *F3H*, and *ANS* genes. However, *ABF2-3* was negatively correlated with the *ANR* gene (Figure 5; Table S7). Thus, *ABF* genes may regulate the expression of downstream genes in response to drought stress.

### 2.5. The Differentially Expressed Transcription Factors Under Drought Stress

**Figure 6:**
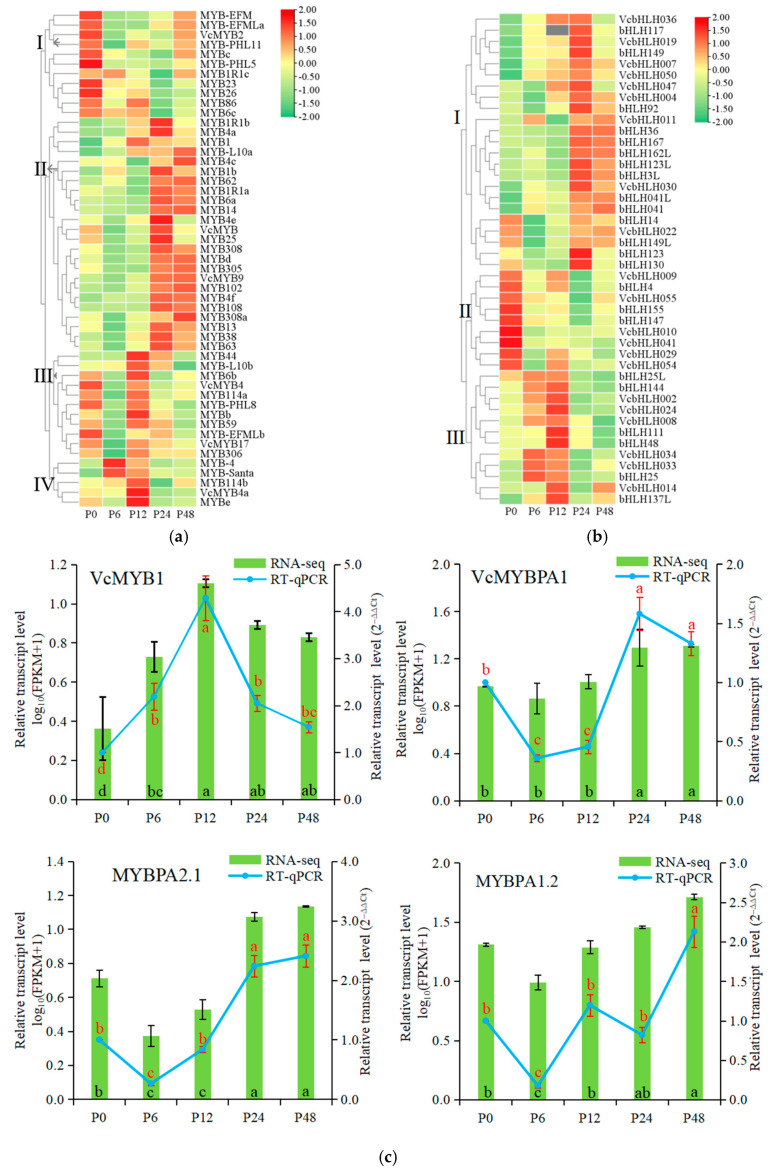

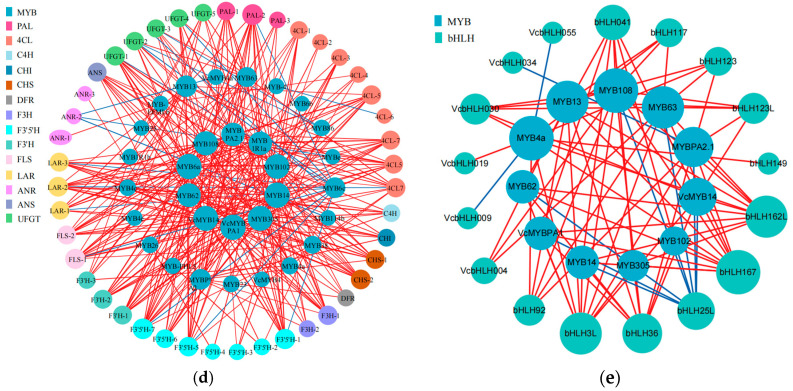
Differentially expressed MYB and bHLH transcription factors in response to drought stress in blueberry leaves by transcriptome deep sequencing (RNA-seq). The heatmap of differentially expressed MYB (**a**) and bHLH (**b**) genes based on log_10_ (FPKM) values. Colored bars on the right indicate low expression (green) or high expression (red) of differentially expressed genes. (**c**) The expression patterns of MYB genes from RNA-seq data based on log_10_ (FPKM + 1) values and RT-qPCR analysis based on 2^−∆∆Ct^ values. Values are means ± SD from three independent biological replicates. Different letters indicate significant differences between samples using *p* value ≤ 0.05. The black error bars and red error bars represent the SD of the samples for RNA-seq and RT-qPCR analysis, respectively. The black letters (a, b, c, and d) and red letters (a, b, c, and d) indicate significant differences between sample for RNA-seq and RT-qPCR analysis, respectively. (**d**) The network of Pearson correlations between MYB genes and differentially expressed flavonoid biosynthetic genes. (**e**) The network of Pearson correlations between MYB and bHLH genes. Red lines indicate positive correlations; blue lines indicate negative correlations. The thickness of the line represents the degree of correlation. The size of the circle represents the number of related genes. P0, P6, P12, P24, and P48 represent that 20% PEG 6000 treated the samples for 0, 6, 12, 24, and 48 h, respectively.

The MYB and bHLH TFs play important roles in response to drought treatment [21,22]. To elucidate the functions of the MYB and bHLH transcription factors in response to drought treatment in blueberry, we identified the differentially expressed MYB and bHLH TFs (Figure 6a,b; Tables S8 and S9). A total of 49 *MYB* genes and 43 *bHLH* genes were differentially expressed in response to drought stress. The MYB genes from clade I were down-regulated, and most *MYB* genes clustered in clade II were up-regulated under drought stress. The expression levels of *MYB* genes from clade III were highest at 0 h and 12 h of drought stress, and from clade IV were highest at 6 h or 12 h of drought stress. Among them, *MYB102*, *MYB108*, *MYB13*, *MYB14*, *MYB305*, *VcMYB14*, *MYB62*, *MYB63*, *MYB4a*, and *VcMYB1* were significantly up-regulated more than four times at 24 and 48 h of drought stress compared to the 0 h control, and *VcMYBPA1*, *MYBPA1.2*, and *MYBPA2.1* also showed higher expression at 24 h and 48 h than the 0 h control and 6 h treatment (Figure 6a; Table S8). RT-qPCR analysis also showed that the expression patterns of *VcMYB1*, *VcMYBPA1*, and *MYBPA1.2* were consistent with the RNA-seq data (Figure 6c). Pearson correlation analysis showed that the expression of *VcMYBPA1*, *MYBPA2.1*, *MYB102*, *MYB108*, *MYB14*, and *VcMYB14* genes positively correlated with almost all the flavonoid biosynthetic genes during drought stress (Figure 6d; Table S10).

For the bHLH TFs, most were clustered in clade I, and drought stress promoted the expression of the *bHLH* genes of clade I. However, drought stress inhibited the expression of the *bHLH* genes of clade II. For the *bHLH* genes of clade III, the expression levels were up-regulated at 6 h or 12 h of drought stress compared to the 0 h control, and then down-regulated at 24 h or 48 h of drought stress. These data showed that most *bHLH* genes were induced by drought treatment (Figure 6b; Table S9). To gain further understanding of the role of the MYB and bHLH TFs under drought stress, the Pearson correlation coefficients (*r*) were calculated between the above thirteen *MYB*s and all the *bHLH*s during drought stress (Figure 6e; Table S11). We found that *bHLH3L*, *bHLH36*, *bHLH167*, *bHLH162L*, *bHLH041*, *bHLH123L*, and *bHLH30* genes are significant correlated with most *MYB* genes. These results indicated that MYBs may regulate the expression of flavonoid biosynthetic genes under drought stress by interacting with bHLH.

### 2.6. Metabolome Analysis for Blueberry Leaves Under Drought Stress

**Figure 7:**
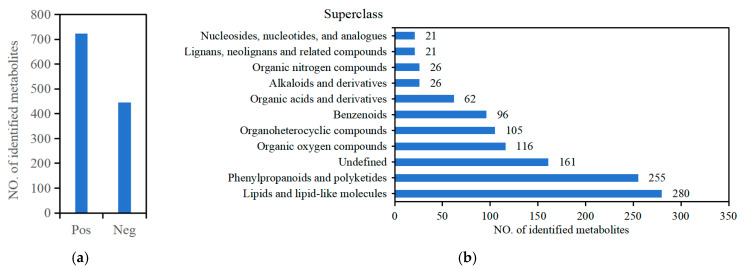
Metabolomic substances were identified by metabolomic profiling in positive and negative ion mode in blueberry leaves in response to drought stress. (**a**) Number of metabolomic substances in positive (Pos) and negative (Neg) ion mode. (**b**) Classification of the detected metabolomic substances.

We detected 1169 metabolomic substances in positive (742) and negative (445) ion mode, including lipids and lipid-like molecules (280), phenylpropanoids and polyketides (255), organic oxygen compounds (116), organoheterocyclic compounds (105), benzenoids (96), organic acids and derivatives (62), alkaloids and derivatives (26), organic nitrogen compounds (26), lignans, neolignans and related compounds (21), and nucleosides, nucleotides, and analogs (21) in blueberry leaves under drought stress (Figure 7a,b; Table S12). The principal component analysis (PCA) showed that QC samples in positive and negative ion modes are closely clustered together and the Pearson correlation coefficient analysis showed that the correlations between QC samples were >0.992 for negative ion mode and 0.978 for positive ion mode, indicating that the metabolome data were reliable (Figures S3 and S4).

### 2.7. Analysis of DAMs for Blueberry Leaves Under Drought Stress

**Figure 8:**
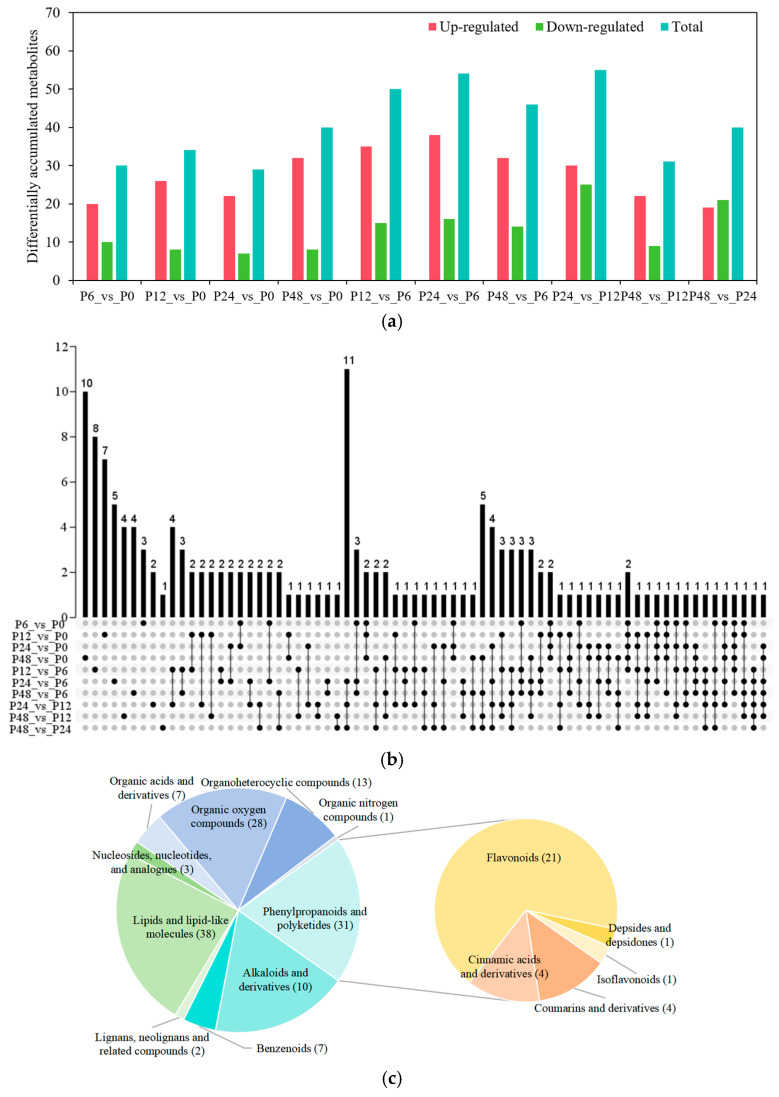
Differentially accumulated metabolites (DAMs) were identified by metabolomic profiling in blueberry leaves in response to drought stress. (**a**) Number of DAMs in response to drought stress. Up-regulated, up-regulated DAMs; Down-regulated, down-regulated DAMs; Total, total DAMs. (**b**) The UpSet Plot shows the number of DAMs for all the pairwise comparisons. (**c**) Classification of the DAMs. P0, P6, P12, P24, and P48 represent that 20% PEG 6000 treated the samples for 0, 6, 12, 24, and 48 h, respectively.

To investigate changes in metabolites under drought stress, the variable importance in the projection (VIP) value of each variable in OPLS-DA was calculated to indicate the contribution of metabolism to the classification. A total of 159 DAMs were identified during drought stress according to VIP value > 1, FC > 1.5, or FC < 0.67 and *p* value < 0.05 (Figure 8a; Table S13). Of these, 30, 34, 29, and 40 DAMs were identified after 6, 12, 24, and 48 h of drought stress relative to the 0 h control samples, respectively. There are the most DAMs at P24_vs_P12 (55 DAMs), and then P24_vs_P6 (54 DAMs) in all the pairwise compared groups under drought stress. At the same time, more DAMs were up-regulated than down-regulated for all the pairwise compared groups (Figure 8a; Table S14). The UpSet Plot showed that specific DAMs were found in each pairwise compared group; for example, 10 DAMs were specific to P48_vs_P0 and 8 to P12_vs_P6 in all the pairwise compared groups (Figure 8b).

These DAMs mainly come from eight superclasses, including lipids and lipid-like molecules (38 DAMs), phenylpropanoids and polyketides (31 DAMs), organic oxygen compounds (28 DAMs), organoheterocyclic compounds (13 DAMs), organic acids and derivatives (7 DAMs), benzenoids (7 DAMs), nucleosides, nucleotides, and analogs (3 DAMs), lignans, neolignans, and related compounds (2 DAMs), and organic nitrogen compounds (1 DAM). Then, we analyzed DAMs from the phenylpropanoid and polyketide superclass and found that flavonoid metabolites (21 DAMs) were the main DAMs and represented the largest class in blueberry leaves under drought stress (Figure 8c). The heatmaps in both positive and negative ion modes revealed that these DAMs were significantly up-regulated or down-regulated under drought stress (Figure S5). For lipids and lipid-like molecule metabolites, most DAMs clustered in clade II were up-regulated, with the highest expression at 12 h or 48 h of drought stress. On the contrary, the expression levels of four DAMs belonging to clade I were lowest at 12 h under drought stress. For phenylpropanoid and polyketide metabolites, the DAMs were down-regulated in clades I and III and up-regulated in clades II and IV. The DAMs were down-regulated in clades I and up-regulated in clades II for organic oxygen compounds. For the other six superclasses, most DAMs were up-regulated under drought stress. To sum up, most DAMs were up-regulated under drought stress.

### 2.8. The DAMs in Flavonoid Biosynthetic Pathways Under Drought Stress

**Figure 9:**
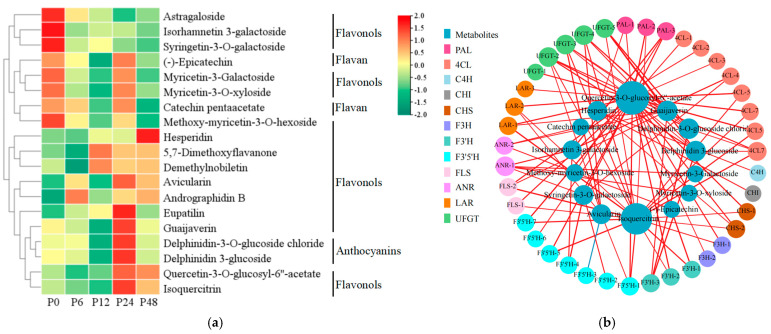
Differentially accumulated flavonoid metabolites and correlation with the differentially expressed flavonoid biosynthetic genes in blueberry leaves in response to drought stress by integrative analysis of transcriptomic and metabolomic data. (**a**) The heatmap of differentially accumulated flavonoid metabolites based on log_10_ (peak intensity) values. Colored bars on the right indicate low expression (green) or high expression (red). (**b**) The network of Pearson correlation between DEGs and DAMs from the flavonoid metabolite pathway. The thickness of the line represents the degree of correlation. The size of the circle represents the number of related genes. P0, P6, P12, P24, and P48 represent that 20% PEG 6000 treated the samples for 0, 6, 12, 24, and 48 h, respectively.

Metabolomic analyses indicated that most DAMs belonged to the flavonoid class; thus, we analyzed the differentially accumulated flavonoid metabolites (flavonols, flavans, and anthocyanins) in response to drought stress (Figure 9a; Table S15). Of these flavonoid metabolites, astragaloside, isorhamnetin 3-galactoside, and syringetin-3-O-galactoside were down-regulated under drought stress. (-)-epicatechin and catechin pentaacetate belong to flavans and also were down-regulated at 6 h or 12 h of drought stress compared to 0 h control, and then up-regulated at 24 h; similar results were found for myricetin-3-galactoside, myricetin-3-O-xyloside, and methoxy-myricetin-3-O-hexoside. Anthocyanins (delphinidin 3-glucoside and delphinidin-3-O-glucoside chloride) and flavonols (quercetin-3-O-glucosyl-6′‘-acetate, guaijaverin, avicularin, isoquercitrin, andrographidin B, hesperidin, eupatilin, 5,7-dimethoxyflavanone, and demethylnobiletin) were up-regulated and the highest accumulation was at 24 h of drought stress. Of these, delphinidin 3-glucoside and delphinidin-3-O-glucoside chloride increased by 8.45- and 7.74-fold at 24 h of drought stress compared to the 0 h control. Thus, most differentially accumulated flavonoid metabolites were up-regulated and were rapidly accumulated at 24 h during drought stress.

### 2.9. Combined Metabolome and Transcriptome Analysis of the Flavonoid Biosynthetic Pathway Under Drought Stress

To investigate the possible relation between the differentially expressed flavonoid biosynthetic genes and differentially accumulated flavonoid metabolites under drought stress, we calculated the Pearson correlation coefficients (*r*) between the FPKM values of the differentially expressed flavonoid biosynthetic genes and peak areas of differentially accumulated flavonoid metabolites (Table S16; Figure 9b). The correlation analysis showed that (-)-epicatechin and catechin pentaacetate (flavan metabolites) were positively correlated with *ANR-2*. Delphinidin 3-glucoside and delphinidin-3-O-glucoside chloride (anthocyanins metabolites) were also positively correlated with *UFGT*s, *4CL7*, and *F3′H-3*. Isoquercitrin and quercetin-3-O-glucosyl-6′‘-acetate (flavonol metabolites) were positively correlated with almost all the flavonoid biosynthetic genes (*PAl*s, *4CL*s, *4CL*5, *4CL*7, *CHS*s, *F3H*s, *F3′5′H*s, *F3′H*s, and *FLS*s). For other flavonoid metabolites, isorhamnetin 3-galactoside and syringetin-3-O-galactoside were positively correlated with *PAL-3*, *4CL-3*, and *F3′5′H-4*, hesperidin was positively correlated with *PAL-3*, *4CL-3*, *C4H*, *CHI*, and *CHS-2*, avicularin with *F3′5′H-1*, methoxy-myricetin-3-O-hexoside with *PAL-3*, and guaijaverin with *F3′H-3*. These data indicated that up- or down-regulation of flavonoid biosynthetic gene expression levels affected accumulations of flavonoid metabolites in response to drought stress.

## 3. Discussion

### 3.1. The DEGs and DAMs in Response to Drought Stress in Blueberry Leaves

Blueberry is especially prone to adverse effects from drought events, and the leaves of blueberry shrink and turn red under drought stress [3]. Blueberry responses to drought stress are accompanied by changes in the expression patterns of numerous genes. Wang et al.’s (2022) [26] study showed that a total of 13,165 DEGs were identified in 3-month-old blueberry cultivar ’Bluecrop’ leaves under 55–60% and 30–35% soil water contents compared to 75–80% soil water contents by the transcriptome profile. Meanwhile, KEGG pathway enrichment analysis showed that most core genes in the leaves in the plant hormone signal transduction pathway and phenylpropanoid biosynthetic pathway and flavonoid biosynthetic pathway were significantly enriched. The *VcABR1*, *VcABF2*, *VcMYB108*, and *VcMYB93* TFs are likely involved in drought response and *VcCYP75A1* are likely involved in anthocyanin biosynthesis under drought stress. In this study, we treated six-month-old blueberry cultivar ’Northland’ plants with 20% PEG-6000 for 0, 6, 12, 24, and 48 h, and detected 14,594 DEGs in blueberry leaves for all the pairwise compared groups. We also found that the plant hormone signal transduction pathway, phenylpropanoid biosynthetic pathway, and flavonoid biosynthetic pathway were significantly enriched under drought stress (Figure 1 and Figure 2). Similar results were found in other plant species under drought stress [27,28,29]. At the same time, we screened the genes from the plant hormone signal transduction pathway, phenylpropanoid biosynthetic pathway, and flavonoid biosynthetic pathway. For example, flavonoid biosynthetic genes including *4CL-2*, *4CL-5*, *4CL-7*, *4CL5*, *4CL7*, *CHI*, *F3H-1*, *F3′5′H-1*, *F3′5′H-5*, *F3′5′H-7*, *F3′H-1*, *F3′H-2*, *FLSs*, *LARs*, and *UFGTs* were up-regulated under drought stress; ABA signal transduction pathway genes, including *PYL-1*, *SnRK2*, *ABF2*, and most *PP2C* genes, were also up-regulated during drought treatment. These genes maybe play an important role in response to drought stress.

For MYB TFs, Wang et al. (2021) [30] observed that a total of 72 *VcMYB* genes were differentially expressed in response to drought stress in blueberry leaves and *VcMYB2*, *VcMYB8*, *VcMYB14*, *VcMYB48*, *VcMYB102*, *VcMYB100*, *VcMYB108*, *VcMYB193*, *VcMYB227*, and *VcMYB228* probably play crucial roles in ROS accumulation and elimination under drought stress. Our study showed that 49 *MYB* genes were differentially expressed in blueberry leaves during drought stress, and *MYB102*, *MYB108*, *MYB13*, *MYB14*, *MYB305*, *VcMYB14*, *MYB62*, *MYB63*, *MYB4a*, and *VcMYB1* were significantly up-regulated more than four times at 24 and 48 h of drought stress compared to the 0 h control. The differences between our study and Wang et al.’s (2021) [30] study may be caused by different experimental varieties and treatment methods; however, both studies showed that *VcMYB14*, *MYB14*, *MYB102*, and *MYB108* were significantly up-regulated under salt stress. In *Vitis vinifera*, MYB14 confers drought tolerance [31]. In *Arabidopsis*, *AtMYB108* is involved in abiotic stress response and *AtMYB102* is up-regulated by drought stress [32,33]. Thus, *VcMYB14*, *MYB14*, *MYB102*, and *MYB108* may be responsible for blueberry leaf drought tolerance.

Drought stress is a major abiotic stress that triggers the accumulation of metabolites [34]. For example, the levels of lipids and lipid-like molecules, phenylpropanoid and polyketides, and organic oxygen compounds were significantly altered in mulberry (*Morus alba*) leaves under drought stress [35]. Drought stress also promoted the accumulation of flavonoid metabolites in many plant species [23,36,37]. Here, we demonstrated that drought stress also affects the accumulation of metabolites in blueberry leaves. These DAMs mainly come from lipids and lipid-like molecules, phenylpropanoids and polyketides, and organic oxygen compounds. Of these, flavonoid metabolite are the main DAMs for the phenylpropanoid and polyketide superclass (Figure 8). The contents of flavonoids and flavonols can be used as indicators for the comprehensive evaluation of the drought tolerance of Chinese chestnut, and flavonoids improve the drought tolerance of maize seedlings [38,39]. Thus, flavonoids play important roles in the tolerance of blueberry to drought stress. Our study also found that flavonoid metabolites including delphinidin 3-glucoside, delphinidin-3-O-glucoside chloride, quercetin-3-O-glucosyl-6′‘-acetate, guaijaverin, avicularin, isoquercitrin, andrographidin B, hesperidin, eupatilin, 5,7-dimethoxyflavanone, and demethylnobiletin were up-regulated under drought stress. Especially, delphinidin 3-glucoside and delphinidin-3-O-glucoside chloride rapidly increase at 24 h in response to drought stress and may be the most important drought-responsive metabolites.

### 3.2. Drought Stress Promotes the Expression of Flavonoid Biosynthetic Genes

Drought stress induces the biosynthesis of flavonoid metabolites by regulating the expression of flavonoid biosynthetic genes [25]. *PAL*, *C4H,* and *4CL*, the genes of the phenylpropanoid biosynthetic pathway, were induced in response to drought stress, leading to a switch to the biosynthesis of other secondary metabolites including flavonoid metabolites [27,40,41]. *CHS*, *F3H*, *F3′H*, *F3′5′H*, and *DFR* were the early genes of flavonoid biosynthesis [28,42]. It has been demonstrated that these genes also play an important role in enhancing drought stress in plants. For example, heterologous expression of the *IbC4H* gene from sweet potato (*Ipomoea batatas*) in tobacco significantly improved drought stress tolerance in transgenic tobacco plants; the overexpression of the *Gh4Cl7* gene of cotton (*Gossypium hirsutum*) in *Arabidopsis* improved tolerance to drought treatment; the overexpression of the *Lycium chinense LcF3H* gene increased the contents of catechin, epicatechin, and epigallocatech and the tolerance to drought stress [43,44,45]. In the current study, most DEGs were up-regulated during drought stress, in which *PAL-1*, *PAL-2*, *C4H*, *4CLs*, *DFR*, and *F3H-2* were down-regulated at 6 h of drought stress, and then were up-regulated at 12 h. The highest expression of these DEGs occurred at 24 h and 48 h of drought stress (Figure 3; Table S5). At the same time, several genes (one *PAL*, two *4CL*, and three *F3′5′H* genes) were down-regulated in response to drought stress. Similar to other plant studies, these DEGs are up-regulated or down-regulated in response to drought stress in blueberry leaves, in which up-regulated genes appear to be predominant in response to drought. Thus, these up-regulated genes may play an important role in promoting flavonoid accumulation and enhancing drought resistance.

FLS is the key enzyme of flavonol biosynthesis, and overexpression of the *FLS* gene increases drought tolerance in plants [46]. In *Brassica rapa*, a total of six *BrFLSs* were identified, and two *BrFLSs* were up-regulated, one *BrFLS* was down-regulated, and three *BrFLSs* were not observed to change significantly under drought stress conditions [47]. The overexpression of *Euphorbia kansui EkFLS* in *Arabidopsis* induces an increase in flavonoid content, improving antioxidant properties and promoting plant resistance to drought and salt stresses [16]. The key genes of flavan biosynthesis *ANR* and *LAR*, and the key genes of anthocyanin biosynthesis *ANS* and *UFGT*, were up-regulated or down-regulated under drought stress [27,28,48]. Here, we found that all the *FLS*, *LAR*, and *UFGT* genes were up-regulated in response to drought stress. *ANS* was down-regulated at 6 h and up-regulated at 24 h and 48 h of drought stress. For three *ANR* genes, *ANR-1* was down-regulated and *ANR-3* was up-regulated under drought stress, and *ANR-2* was down-regulated at 12 h and up-regulated at 24 h of drought stress (Figure 3; Table S5). These results indicate that drought stress up-regulated the expression of most flavonoid biosynthetic genes.

### 3.3. The Regulatory Network of Drought Stress-Induced Accumulation of Flavonoid Metabolites in Blueberry Leaves

**Figure 10:**
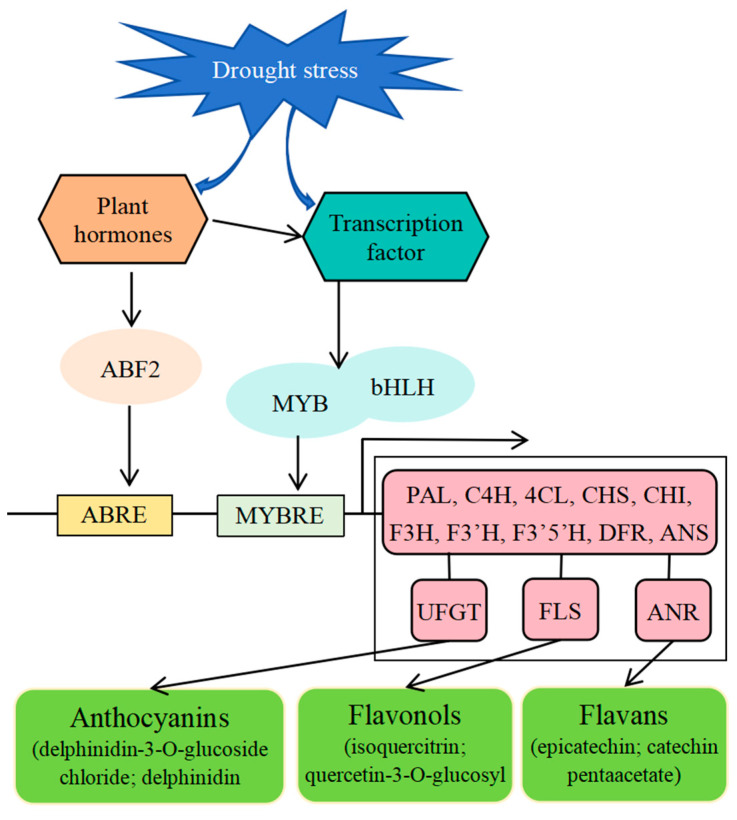
The regulatory network of drought stress-induced accumulation of flavonoid metabolites in blueberry leaves by integration of transcriptomic and metabolomic data.

Plant hormones are also involved in drought stress responses in plants. In blueberry, the pathway with the most core genes in leaves is the plant hormone signal transduction pathway under drought [26]. Our study also showed that plant hormone pathways (ABA, GA, Auxin, SA, BR, CT, JA, and ET) were enriched under drought stress in blueberry leaves. Of these, the ABA signaling pathway is central to drought stress responses in plants [8]. ABA also promotes the accumulation of anthocyanin in blueberry leaves [49]. In general, ABA induces gene expression by binding to conserved cis-acting ABREs in the promoters of their target genes. ABF transcription factors regulate the ABRE-mediated transcription of downstream target genes [50,51,52]. For example, PeABF3 activates the expression of *actin-sepolymerizing factor-5* (*PeADF5*) by directly binding to its promoter to enhance drought tolerance in *Populus euphratica* [25]. *Actinidia chinensis* AchnABF2 and AchnMYB transcription factors regulate expression of *ω-hydroxyacid/fatty alcohol hydroxycinnamoyl transferase* (*FHT*) and accumulation of suberin monomers [53]. Here, three *ABF2* genes were up-regulated under drought stress in blueberry leaves and were significantly correlated with many flavonoid biosynthetic genes, including *4CL*, *C4H*, *CHS*, *DFR*, *F3H*, *F3′5′H*, *FLS*, *LAR*, *ANR*, *ANS*, or *UFGT* genes (Figure 5). Thus, drought stress up-regulated the expression of *ABF2*, and *ABF2* may affect the expression of flavonoid synthesis genes to improve the tolerance of blueberry leaves to drought stress (Figure 10).

In *Arabidopsis*, the R2R3-MYB family members are divided into 25 subgroups [32,54]. *Vaccinium myrtillus* VmMYBPA1.2 (homolog MYBPA1.2) and VmMYBPA2.1 (homolog MYBPA2.1) belong to subgroup 5 and induced the accumulation of anthocyanin, and VcMYBPA1 may be a regulator of proanthocyanidin biosynthesis [55,56]. VcMYB1 promoted anthocyanin synthesis in *Arabidopsis*, tobacco plants, and green blueberry fruits [57]. At the same time, these MYB TFs were up-regulated and positively correlated with almost all the genes of the flavonoid biosynthetic pathway. Thus, these MYB TFs might be responsible for blueberry leaf drought tolerance, and VcMYB1, VcMYBPA1, MYBPA1.2, and MYBPA2.1 may be involved in drought stress-induced flavonoid biosynthesis by regulating the expression of flavonoid biosynthetic genes (Figure 10).

The bHLHs comprise one of the largest families of TFs in plants. They have been shown to be involved in responses to various abiotic stresses [58]. In *Arabidopsis*, both AtbHLH122 and AtbHLH112 function as a positive regulator of drought stress [59,60]. The heterologous expression of the *Anthurium andraeanum AabHLH35* gene in *Arabidopsis* improved tolerance to drought stresses [61]. At the same time, bHLH TF also regulates flavonoid biosynthesis. For example, the grape *VvbHLH1* gene increases the accumulation of flavonoids and enhances drought tolerance in transgenic *Arabidopsis* [22]. However, most studies have shown that MYB-bHLH interactions are responsible for flavonoid biosynthesis by regulating the gene expression of the flavonoid biosynthesis pathway. In grape, bHLH TF VvMYC1 cannot activate the promoters of *CHI*, *UFGT*, and *ANR* genes in the flavonoid biosynthetic pathway by itself, but the co-transfection of *VvMYC1* with a *MYB* transcription factor can significantly activate the expression of *CHI*, *UFGT*, and *ANR* genes [62]. In *Actinidia chinensis*, *AcMYB123* and *AcbHLH42*, two interacting transcription factors, regulate tissue-specific anthocyanin biosynthesis in the inner pericarp [63]. In *Populus deltoids*, MYB transcription factor PdMYB118 directly interacts with bHLH transcription factor PdTT8 to regulate wound-induced anthocyanin biosynthesis [64]. Here, 43 bHLH TFs responded to drought stress and 17 bHLH TFs were significant correlated with MYB TFs during drought stress. Thus, MYB transcription factors may regulate expression of flavonoid biosynthesis genes by interacting with bHLH proteins under drought stress (Figure 10).

Flavonoid metabolites are mainly composed of anthocyanins, flavonols, flavans, and proanthocyanidins. In tobacco, the accumulation of anthocyanins conferred a higher drought tolerance to the plant [65]. In *Arabidopsis*, the accumulation of anthocyanins and flavonols increases reactive oxygen species scavenging and activates other stress responses, such as osmotic adjustment and ion transport, to improve tolerance to drought stresses [66]. In *Cistus clusii*, drought induced the accumulation of epicatechin [67]. In this study, drought stress promoted the accumulation of anthocyanins (delphinidin-3-O-glucoside chloride and delphinidin 3-glucoside), and most flavonols (isoquercitrin, quercetin-3-O-glucosyl-6′‘-acetate, and so on). At the same time, flavans (epicatechin and catechin pentaacetate) were down-regulated at 12 h and 48 h compared to 6 h of drought stress and up-regulated at 24 h compared to 6 h and 12 h of drought stress (Figure 9; Table S15). Pearson correlation coefficient analysis showed that the expression of four *UFGT* genes was positively correlated with the accumulation of delphinidin-3-O-glucoside chloride and delphinidin 3-glucoside, and the expression of two *FLS* genes was positively correlated with the accumulation of isoquercitrin and quercetin-3-O-glucosyl-6′‘-acetate during drought stress. Furthermore, *ANR-2* was positively correlated with epicatechin and catechin pentaacetate. At the same time, the expression of *PAl*, *4CL*, *4Cl5*, *4CL7*, *CHS*, *F3H*, and *F3′5′H* was also positively correlated with the accumulation of isoquercitrin and quercetin-3-O-glucosyl-6′‘-acetate during drought stress. The *PAl*, *4CL*, *4Cl5*, *4CL7*, *CHS*, *F3H*, and *F3′5′H* genes were the main genes of the flavonoid biosynthetic pathway (Figure 9b; Table S16). UFGT, FLS, ANR were the last enzymes in biosynthesis of anthocyanins, flavonols, and flavans, respectively [48,68]. Thus, four *UFGT* genes may be the key genes for anthocyanin (delphinidin-3-O-glucoside chloride and delphinidin 3-glucoside) biosynthesis, two *FLS* genes may be responsible for the accumulation of flavonols (isoquercitrin and quercetin-3-O-glucosyl-6′‘-acetate), and *ANR-2* may be a key gene for flavan (epicatechin and catechin pentaacetate) biosynthesis during drought stress (Figure 10).

## 4. Materials and Methods

### 4.1. Plant Materials and Drought Stress Treatments

The in vitro-grown blueberry cultivar ‘Northland’ plants were kept in the laboratory at Department of Horticulture of Jilin University, China. The in vitro-grown plants were transferred to 7 cm pots containing soil and grown in a growth chamber at 25 °C with 70% relative humidity under a 16 h light (from 6:00 to 22:00)/8 h dark (from 22:00 to 6:00) photoperiod for six months. The potted plants were irrigated with 1/2 Hoagland solution containing 20% polyethylene glycol 6000 (PEG 6000) [42,69]. The PEG 6000 was used for irrigation at 8:00 (it had been light for 2 h) when the relative soil water content was 75–80%. For each treatment, the first to fifth fully expanded leaves were collected from ten randomly selected plants (as one biological replicate) at 6 h (P6), 12 h (P12), 24 h (P24), and 48 h (P48) of PEG 6000 treatment, without PEG 6000 treatment at 8:00 as the 0 h (P0) control. The leaves were flash-frozen in liquid nitrogen and stored at −80 °C for transcriptome deep sequencing (RNA-seq) analysis and metabolomic profiling. RNA-seq analysis was performed on three replicates, while the metabolomic profiling experiment was performed on five replicates.

### 4.2. Transcriptomic Analysis by RNA Sequencing

Total RNA was extracted from each sample using TRIzol^®^ Reagent (Invitrogen, Waltham, MA, USA), and paired-end libraries were prepared using an ABclonal mRNA-seq Lib Prep Kit (ABclonal, Wuhan, China) following the manufacturer’s instructions. The libraries were sequenced on a DNBSEQ-T7 instrument, and 150 bp paired-end reads were generated. Raw data in fastq format were processed using in-house perl scripts. The clean reads were used for subsequent analysis by removing the adapter sequences and filtering out low-quality reads and reads with N ratios > 5%. The GC content of clean reads and a quality score of Q20 and Q30 were calculated to evaluate base quality. The clean reads were separately aligned to the reference *Vaccinium corymbosum* cv. Draper V1.0 genome sequence (https://www.vaccinium.org/genomes, accessed on 25 March 2024) with orientation mode using HISAT 2.2.1 software (http://daehwankimlab.github.io/hisat2/, accessed on 25 March 2024) to obtain mapped reads.

FeatureCounts (http://subread.sourceforge.net/, accessed on 5 April 2024) was used to count the number of reads mapped to each gene. Quantification of gene expression levels was estimated by the fragments per kilobase of transcript per million fragments mapped (FPKM) values. Differential expressed genes (DEGs) from all pairwise compared groups were identified based on the criteria of absolute log_2_ fold change (FC) ≥ 1 and *p* value < 0.05 using DESeq2 (http://bioconductor.org/packages/release/bioc/html/DESeq2.html, accessed on 10 April 2024). KEGG pathway statistical enrichment analysis of DEGs was implemented using KOBAS 3.0 software.

### 4.3. RNA-seq Data Validation

The RT-qPCR was performed on an ABI 7900HT real-time PCR system. Ten genes of interest (*4CL-1*, *4CL-3*, *4CL-5*, *PAL-1*, *PAL-3*, *CHS-1*, *F3H-1*, *MYB114a*, *ABF2-1*, *PYL1*, and *ABF2-1*) from the flavonoid biosynthetic pathway, MYB TFs, or plant hormone signal transduction pathway were selected to validate the accuracy and reliability of the RNA-seq data. The expression levels of *VcMYB1*, *VcMYBPA1*, *MYBPA2.1*, *MYBPA1.2*, *UFGT-2*, *UFGT-3*, *UFGT-4*, *UFGT-5*, *FLS-1*, *FLS-2*, and *ANR-2* genes were used for RT-qPCR analysis. Glyceraldehyde-3-phosphate dehydrogenase (*GAPDH*; GenBank accession no. AY123769) was used as the reference transcript. Primer information is presented in Table S17. The relative expression levels of each gene were calculated using the 2^−∆∆Ct^ method.

### 4.4. Metabolomics Analysis by UHPLC-MS/MS

For metabolite extraction, each powdered sample (80 mg) was added in 1 mL methanol/acetonitrile/H_2_O (2:2:1, *v/v/v*) solution, and then centrifuged at 4 °C at 14,000× *g* for 20 min. The supernatant was dried in a vacuum centrifuge. For LC–MS analysis, the samples were re-dissolved in 0.1 mL acetonitrile/water (1:1, *v/v*) solvent and centrifuged at 4 °C at 14,000× *g* for 15 min, and then the supernatant was placed in an automatic sampler. Ultra-high performance liquid chromatography–quadrupole time-of-flight mass spectrometry (UHPLC-Q-TOF-MS) was performed using an Agilent 1290 infinity LC ultra-performance liquid chromatography (UHPLC) system with a C-18 column (ACQUITY UPLC BEH C-18 1.7 μm, 2.1 mm × 100 mm; Waters, Ireland) coupled to a quadrupole time-of-flight instrument (AB Sciex Triple TOF 6600). Mobile phase A consisted of 25 mM ammonium acetate and 0.5% formic acid in water, and mobile phase B was methanol. The gradient elution procedure was as follows: 5% methanol (0–0.5 min); methanol changed linearly to 100% (0.5–10 min); 100% methanol (10–12 min); methanol changed linearly from 100% to 5% (12.0–12.1 min); and then 5% methanol (12.1–16 min). The temperature of the column was maintained at 40 °C and the flow rate was 0.4 mL/min. Quality control (QC) samples were inserted into the sample queue to monitor and evaluate the stability and reliability of the data.

For metabolome data analysis, the raw data were converted to the final data format by employing ProteoWizard MSConvert (http://proteowizard.sourceforge.io, accessed on 5 June 2024), and the matched peak data and peak area data were obtained using MS-DIAL software (ver. 4.60) for normalization. After being normalized to total peak intensity, the processed data were analyzed by an R package, where they were subjected to multivariate data analysis, including Pareto-scaled principal component analysis (PCA) and orthogonal partial least-squares discriminant analysis (OPLS-DA). Seven-fold cross-validation and response permutation testing were used to evaluate the robustness of the model. The variable importance in the projection (VIP) value of each variable in the OPLS-DA model was calculated to indicate its contribution to the classification. Metabolites with a VIP value > 1 were further subjected to Student’s t-test at the univariate level to measure the significance of each metabolite; *p* values less than 0.05 were considered as statistically significant.

### 4.5. Data Processing and Statistical Analysis

Pearson correlation coefficients were calculated to determine the correlation between DEGs and between differentially expressed flavonoid biosynthetic genes and differentially accumulated flavonoid metabolites using SPSS 19.0 software. Heatmaps of gene expression levels were constructed using log_10_ (FPKM), and heatmaps of metabolites were constructed using log_10_ (peak intensity) values with Tbtools (v1.098761) software [70]. For RT-qPCR analysis, all experiments were carried out with three independent biological replicates, and three technical replicates were performed for each biological replicate.

## 5. Conclusions

In this study, we conducted transcriptomic analysis by RNA-seq and metabolomic analysis via UHPLC-MS/MS to explore the underlying molecular mechanism of flavonoid biosynthesis in response to drought stress for blueberry leaves. We found that drought stress promoted the expression of most flavonoid biosynthetic genes and the accumulation of most flavonoid metabolites during drought stress. *UFGTs*, *FLS*s, and *ANR* may be the key genes for drought-induced anthocyanin, flavonol, and flavan biosynthesis in blueberry leaves, respectively. Anthocyanins (delphinidin 3-glucoside and delphinidin-3-O-glucoside chloride) may be the most important drought-responsive flavonoid metabolites and *VcMYB1*, *VcMYBPA1*, *MYBPA1.2*, *MYBPA2.1*, *VcMYB14, MYB14, MYB102*, *and MYB108* might be important drought-responsive transcription factors in blueberry leaves. Pearson correlation analysis showed that the regulatory network encompassing *ABFs*, *MYBs*, *bHLHs*, and flavonoid biosynthetic genes may regulate drought-induced accumulation of flavonoid metabolites in blueberry leaves. In this study, the identification of drought-responsive genes and metabolites will help to uncover the underlying molecular mechanism of drought tolerance and will be useful for breeding drought-tolerant blueberry cultivars.

## Data Availability

Data are contained within the article and Supplementary Materials. We have uploaded the RNA-Seq data generated in this study to BioProject in the NCBI repository with the accession number of PRJNA1160404.

## Supplementary Materials

The following supporting information can be downloaded at: https://www.mdpi.com/article/10.3390/ijms252011135/s1.
