# Supplementary material for: Integrated Analysis of Transcriptome and Metabolome Provides Insights into Flavonoid Biosynthesis of Blueberry Leaves in Response to Drought Stress
Source: Int J Mol Sci. 2024 Oct 17;25(20):11135. doi: 10.3390/ijms252011135 (PMC11508776; doi:10.3390/ijms252011135)
Supplement: Supplementary file 1 [file ijms-25-11135-s001.zip › Supplement Figures.pdf]

a

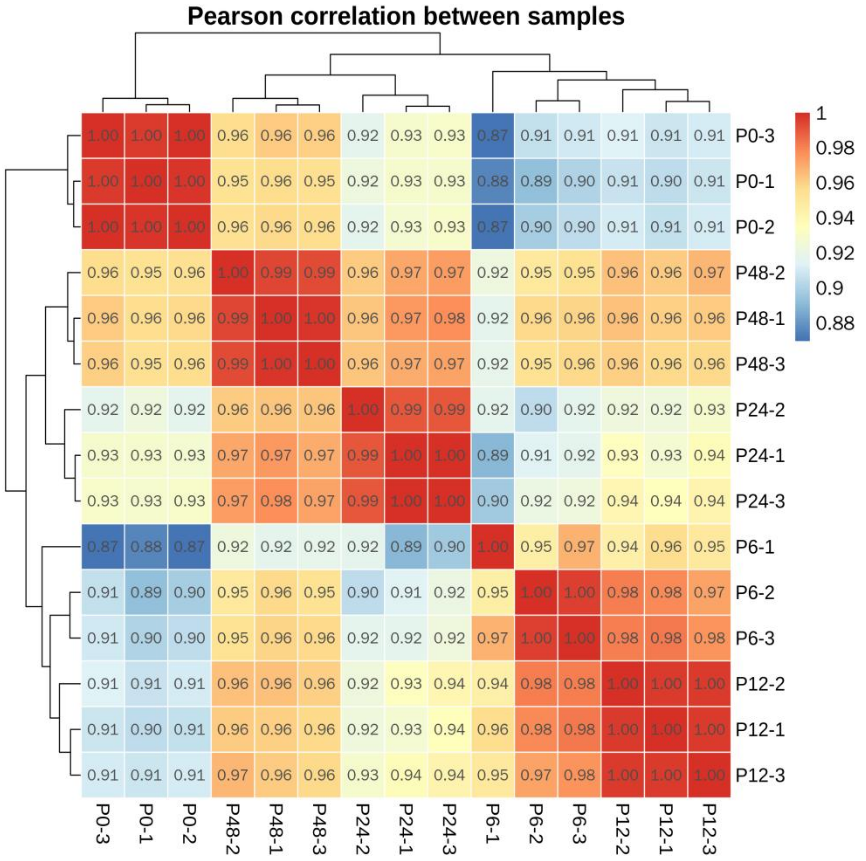

b

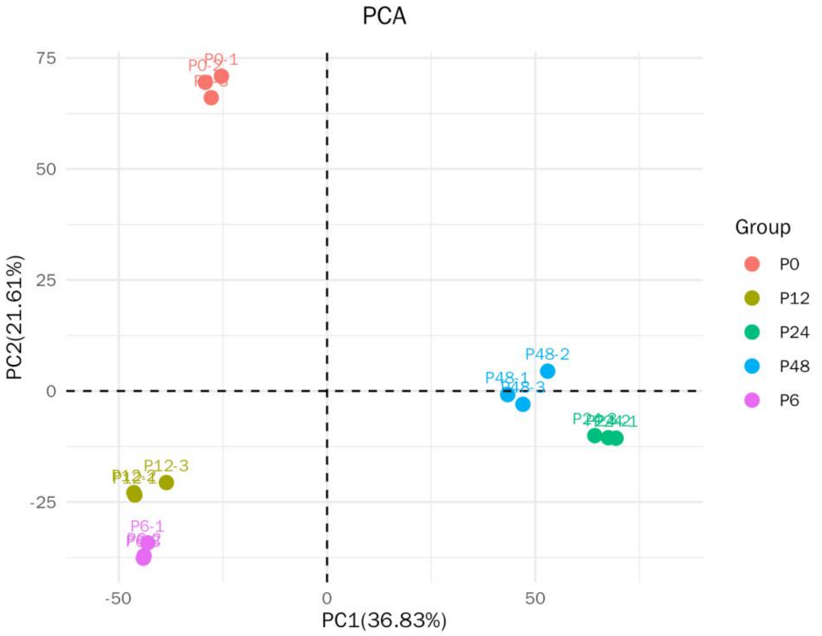

Figure S1 Pearson correlation and principal component analysis (PCA) between experimental samples by transcriptome deep sequencing (RNA-seq) under drought stress. (a) Pearson correlation between samples. (b) PCA between experimental samples. P0, P6, P12, P24, and P48 represent that 20% PEG 6000 treated the samples for 0, 6, 12, 24, and 48 h, respectively.

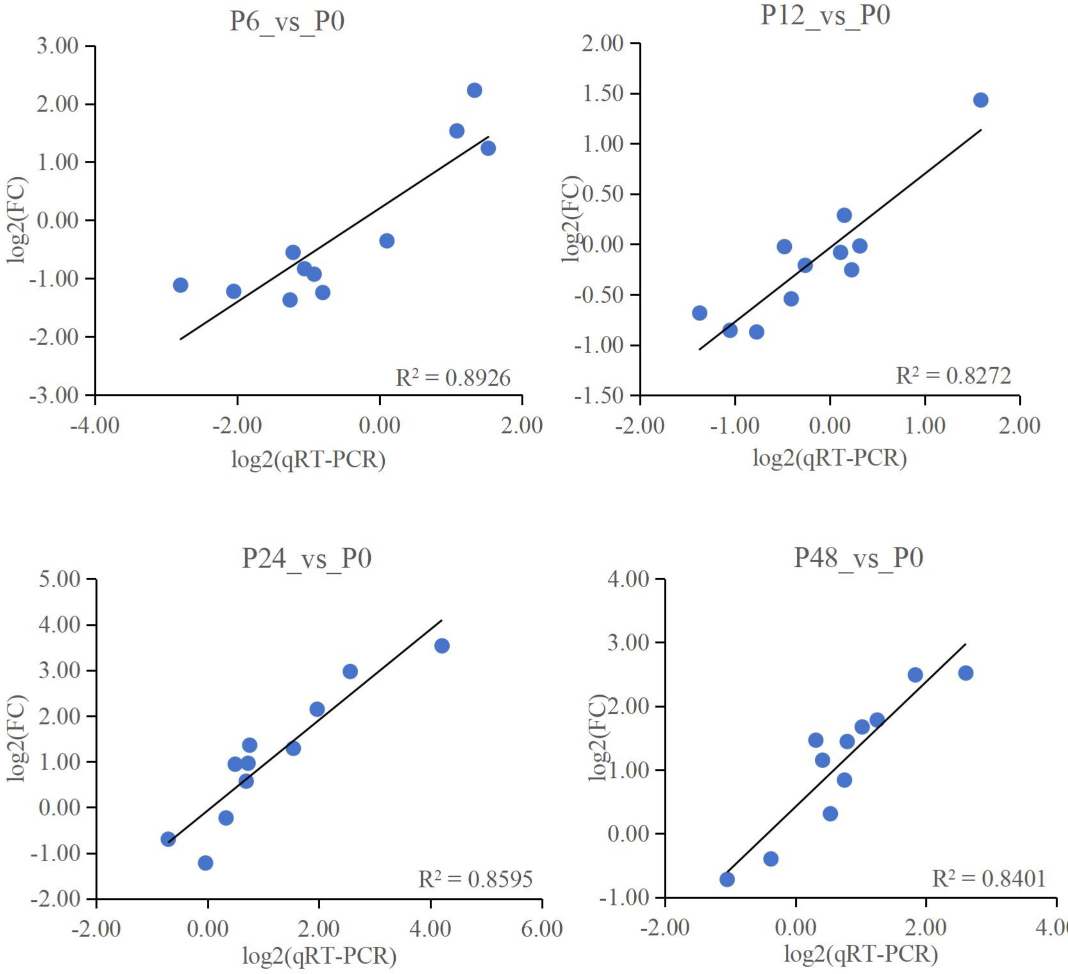

Figure S2 Validation of transcript expression changes by qRT-qPCR. The figure is based on $\log_2(2^{-\Delta\Delta Ct})$ data from qRT-PCR and $\log_2$(fold change) data from RNA-seq. The linear trend line and the R^2 are shown

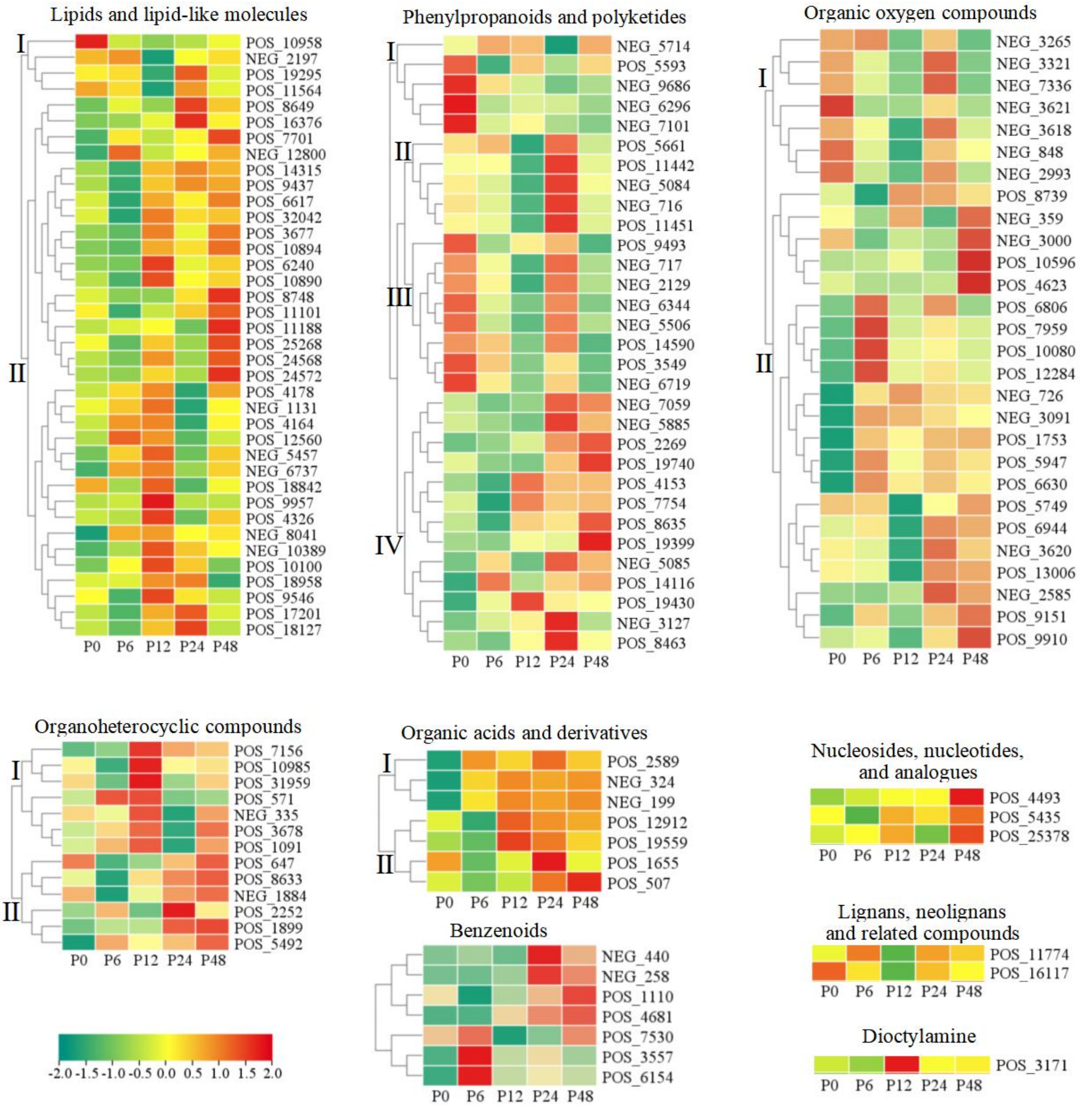

Figure S3 The heatmap of differentially accumulated metabolites in blueberry leaves in response to drought stress by metabolomic data based on log_{10} (peak intensity) values. Colored bars on the bottom left indicate low expression (green) or high expression (red). P0, P6, P12, P24, and P48 represent that 20% PEG 6000 treated the samples for 0, 6, 12, 24, and 48 h, respectively.

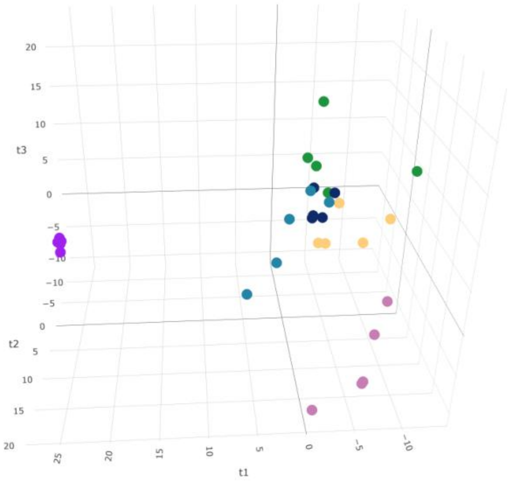

PCA3D negative ion modes

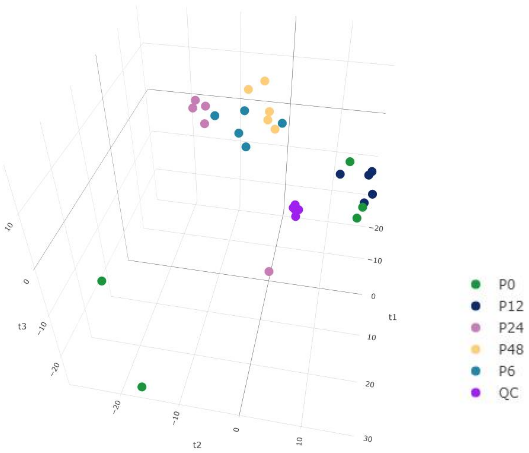

PCA3D positive ion modes

Figure S4 Principal component analysis (PCA) of the peaks obtained from all experimental samples and quality control (QC) samples from by metabolomic data. P0, P6, P12, P24, and P48 represent that 20% PEG 6000 treated the samples for 0, 6, 12, 24, and 48 h, respectively.

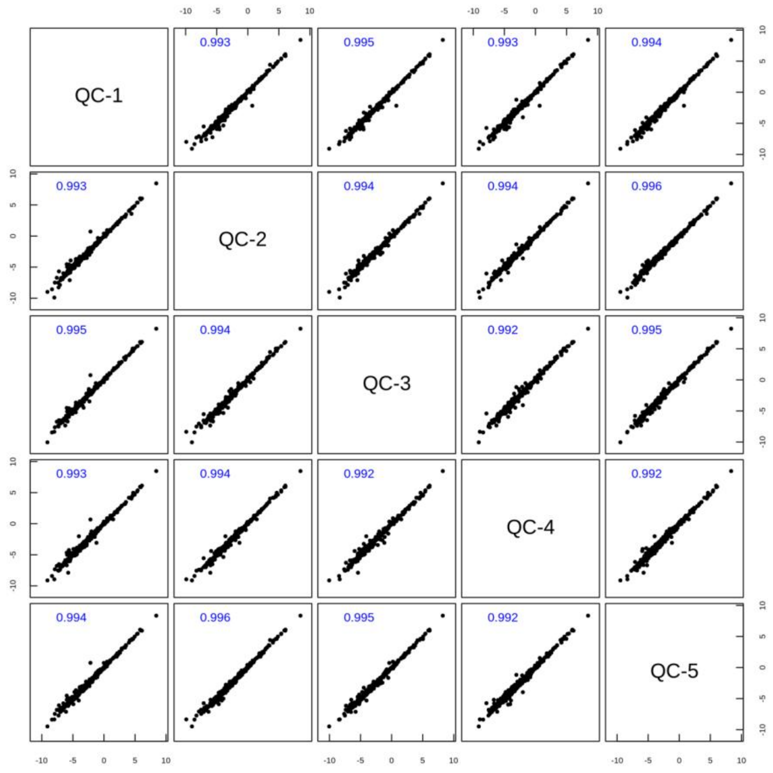

MultiScatter negative ion mode

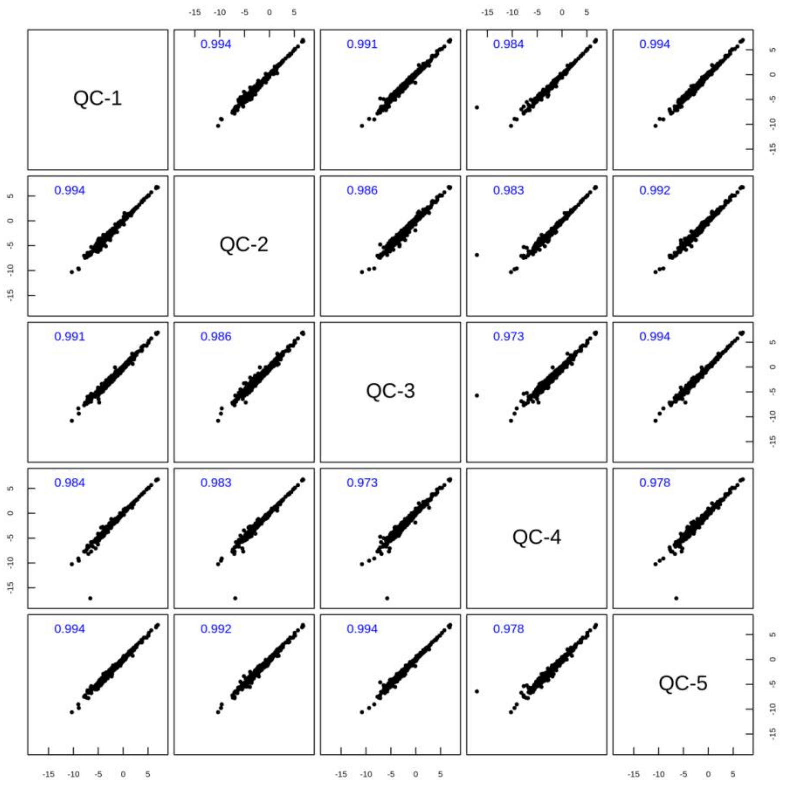

MultiScatter positive ion mode

Figure S5 Pearson correlation coefficients between quality control samples (QC) samples detected in negative ion mode and positive ion mode. The points in each small grid represent the ion peaks (metabolites) extracted from QC samples.
